# DNA methylation of GDF-9 and GHR genes as epigenetic regulator of milk production in Egyptian Zaraibi goat

**DOI:** 10.1007/s13258-023-01464-9

**Published:** 2023-11-20

**Authors:** Layaly Gamal, Magda M. Noshy, A. M. Aboul-Naga, Hussein Sabit, Haidan M. El-Shorbagy

**Affiliations:** 1 Sheep and Goat Research Department, Animal Production Research Institute, Agriculture Research Center (ARC), Giza, Egypt; 2 Zoology Department, Faculty of Science, Cairo University, Giza, 12613 Egypt; 3 Department of Medical Biotechnology, College of Biotechnology, Misr University for Science and Technology, Giza, Egypt; 4 Faculty of Biotechnology, October University for Modern Science and Arts, 6th October, Giza, Egypt

**Keywords:** DNA methylation, GDF-9, GHR, Goat, Milk production, MSP

## Abstract

**Background:**

DNA methylation is an epigenetic mechanism that takes place at gene promoters and a potent epigenetic marker to regulate gene expression.

**Objective:**

The study aimed to improve the milk production of Zaraibi goats by addressing the methylation pattern of two milk production-related genes: the growth hormone receptor **(**GHR**)** and the growth differentiation factor-9 (GDF-9).

**Methods:**

54 and 46 samples of low and high milk yield groups, respectively, were collected. Detection of methylation was assessed in two CpG islands in the GDF-9 promoter via methylation-specific primer assay (MSP) and in one CpG island across the GHR promoter using combined bisulfite restriction analysis (COBRA).

**Results:**

A positive correlation between the methylation pattern of GDF-9 and GHR and their expression levels was reported. Breeding season was significantly effective on both peak milk yield (PMY) and total milk yield (TMY), where March reported a higher significant difference in PMY than November. Whereas single birth was highly significant on TMY than multiple births. The 3rd and 4th parities reported the highest significant difference in PMY, while the 4th parity was the most effective one on TMY.

**Conclusion:**

These results may help improve the farm animals' milk productive efficiency and develop prospective epigenetic markers to improve milk yield by epigenetic marker-assisted selection (eMAS) in goat breeding programs.

## Introduction

Goats are a vital source of milk production, especially in the desert and rural areas in the delta region (Abd-Allah et al. 2019). There are two main local Egyptian goat breeds, raised in the Delta namely; Baladi and Zaraibi (Galal 2005, Galal and Scienoes 2010). Zaraibi Goat (or Egyptian Nubian) is the most local goat in Egypt, especially Northern Delta, due to its efficiency in producing meat and milk at low cost (Capote et al*.* 2016, Moawed and Shalaby 2018). Most dairy products are of high nutritional importance and are processed from ruminant milk (cow, buffalo, and goat) that contains specific bioactive proteins, lipids, saccharides, immunoglobulins, and many other vital components (Kholif et al*.* 2020). The average milk production of the Zaraibi goat is 253.1 kg in the milking season including 90 days of lactation (Soltan, et al. 2021).

Zaraibi goat is the most promising goat among Egyptian breeds that has a high genetic potential for milk production (Galal 2005). Several candidate genes have been revealed through previous genome-wide association studies (GWAS) regarding milk production traits in dairy goats. Growth hormone receptor (GHR) was strongly suggested as a functional gene for milk quality traits (Sanchez et al. 2016). GHR gene mediates most functions of growth hormone (GH) such as mammary gland growth, lactation, and fertility (Lucy 2008). Several studies reported a clear association between GHR polymorphisms and milk production, quality, and coagulation properties (Rahmatalla et al. 2011; Waters et al. 2011; Sanchez et al. 2016; Viale et al. 2017; El-Komy et al. 2020). GDF-9 is another candidate gene that performs a crucial function in the reproduction process through the development and differentiation of ovarian follicles (Tang et al. 2018). It has many polymorphisms that are associated with milk production, milk content, prolificacy, litter size, female fertility, and ovulation (Gorlov, et al. 2018; Al-Khuzai and Ahmed 2019; Koyun et al. 2021; Wang et al. 2021). Moreover, several environmental factors could affect milk production efficiency. Many authors reported a significant influence of parity on peak and total milk yield (Agnihotri and Rajkumar 2007; Pawar et al. 2012; Shaat 2014). In addition, milk production at all phases of lactation are directly affected by litter size, parity number and kidding in dairy goats (Zamuner et al. 2020). Factors like kidding numbers, and kidding season demonstrate their significant effects on milk yield (Maldonado et al*.* 2018). Accordingly, many environmental variables can alter the expression of several genes and result in phenotypic differences without altering the nucleotide sequence of their DNA. These modifications are known as epigenetic changes. These changes may involve amino acid modifications of histone protein where DNA is wrapped, non-coding RNA expression, changes in DNA methylation status, and RNA methylation (Skinner et al. 2010; Mongan et al. 2019). The addition of methyl group to the C5 position of the cytosine ring is called DNA methylation, where cytosine should come before guanine to produce 5-methyl cytosine (5-mc) in the clusters of CpG dinucleotides in the promoter of the gene. CpGs are uncommon in the genome; they are short DNA segments ranging in length from 300 to 3000 base pairs, that’s why they are known as CpG islands. Methylation of promoter sequences prevents some transcription factors from binding to them, so it is considered a powerful epigenetic marker and regulator of gene expression (Cedar and Bergman 2012; Barazandeh et al. 2019). Epigenetic processes can alter gene expression in response to various environmental factors and provide a link between environmental variations and animal physiology (Donkin and Barrès 2018). Recently, the role of epigenetic factors as an additional tool for the genetic regulation of livestock animal traits, management, and productivity has been addressed (Ibeagha and Yu 2021). Several techniques and analyses could discuss the relationship between methylation and gene expression and their association with milk production such as genome-wide DNA methylation, Function enrichment analysis, and methylation-sensitive Single Nucleotide Primer Extension (Ms-SNuPE) (Kurdyukov et al*.* 2014 and Wanting et al*.* 2020). A considerable number of reports have estimated the methylation status of many candidate genes for their correlation with milk production in different livestock (Pauwels et al. 2017; Chen et al. 2018; Zhao et al. 2019). However, little is known about DNA methylation patterns and the expression of milk production genes in goats. The present study aimed to explore the effect of DNA methylation in the promoter region of two milk production-related genes (GDF-9, GHR) on the milk production of the Zaraibi goat breed as an epigenetic marker to improve the productive efficiency of farm animals and investigate potential epigenetic markers to improve milk yield.

## Materials and methods

### Chemicals

All used molecular kits and chemicals were of analytical quality, purchased from Qiagen, Thermo-Scientific, Zymo-Research, and Willow-fort Research Services Co. Cairo, Egypt. All reagents were utilized by the required safety and health protocols.

### Animals and ethical considerations

Handling and protection of animals used in the study were done according to the recommendations of European Union directive 86/609/EEC (Louhimies 2002) and approved by the Animal Production Research Institute (APRI), Agriculture Research Center (ARC), Ministry of Agriculture and Land Reclamation (MALR) with permit number: CUIS8117. The does were maintained under similar management practices and were fed 25% concentrate-fed mixture (CFM), 75% fresh berseem throughout winter, and 50% CFM and 50% berseem in summer (NRC 2007). On El-Serw farm, (APRI) 100 adult female Zaraibi dairy goats were used, and milk samples were collected from each doe. The average body weight of the does was 31.5 kg, and the parity (kidding season of doe) was in the third, fourth, and fifth parities. Their ages ranged from 4 to 7 years. The litter size was recorded by the number of kids born for each doe (single or multiple births). Their breeding seasons were in November and March. Does were divided into low and high-producing animals.

### Genomic DNA extraction and bisulfite treatment

Somatic cells of low and high milk yield groups were collected from 50 mL of milk samples from each goat for genomic DNA extraction using the QIAmp DNA Mini Kit (Qiagen, GmbH, Germany). The concentrations of DNA were measured. 1000 ng of genomic DNA was converted by sodium bisulfate (from cytosine to uracil) using a ZYMO RESEARCH-EZ DNA Methylation-Gold Kit, following the manufacturer’s instructions. Briefly, 130 µL of conversion reagents were added to 20 µL of DNA and incubated at 64 °C for 2.5 h in Zymo-Spin TM IC Column. 600 µL M-binding buffer was added to the Zymo-Spin TM IC Column and centrifuged, then 100 µL of M-wash buffer was added followed by 200 µL of M-Desulphonation buffer, and finally,15 µL of M-elution buffer to elute the methylated DNA. The output was tested by two-direction sequencing to confirm the successfulness of the conversion process.

### Prediction of the CpG island and methylation primer design

A free online tool MethPrimer https://www.urogene.org/methprimer/ was used to identify CpG islands within the promoters of GHR and GDF-9 genes and to design primers used to amplify these CpGs specific regions (Li and Dahiya 2002). Two CpGs islands in the promoter region (840 bp) of the GDF-9 gene were found. Island 1 at region (49 – 309 bp), and Island 2; at region (512 – 615 bp) which was amplified using methylation-specific primers. Regarding the GHR gene promoter (660 bp), there was one CpGs Island found at region (295 – 407 bp) which was amplified using restriction sites primers used for COBRA analysis as shown in Table 1.

**Table 1:** Primer sets for detection of gene methylation pattern

MSP of the GDF-9 Gene
Left M: 5′- GAGGTCGTCGTTTGGTAGTTAAC -3′
Right M: 5′- GTATCCCTAATTCCGATCTTACGAT -3′
Left U: 5′- TTGAGGTTGTTGTTTGGTAGTTAAT-3′
Right U: 5′- CCATATCCCTAATTCCAATCTTACA-3′
Restriction Primers of the GHR Gene
Left: 5′- ATGGAAATAATTTATGTTTGATTGT-3′
Right: 5′- AAAATAACAACCCACTCCAATATTCT-3′

### PCR amplification of bisulfite-treated DNA

The CpG regions within the GHR and GDF-9 genes were amplified using COSMO PCR red master mix kit. The reaction contained about 12.5 µL of 2 × Cosmo PCR master mix, 1.5 µL (10 pmol) from each primer, and 2.5 µL converted DNA, then nuclease-free water up to 25 µL to reach the total reaction volume. The thermal-cycler program was as follows: Initial denaturation at 95 °C for 2 min, 32 cycles of denaturation step at 95 °C for 15 s. then annealing at 60.3 °C, 58.8 °C, and 48 °C for methylated GDF-9, unmethylated GDF-9, and restriction primer of GHR, respectively for 30 s., then extension at 72 °C for 1 min, and finally, the final extension step at 72 °C for 5 min. The methylation pattern was detected after electrophoresing the PCR products in 2.5% agarose and reporting the presence or absence of bands for each primer. Successful PCR products for each CpG region were 114 bp, and 118 bp for methylated and unmethylated patterns of GDF-9, respectively while the PCR product of GHR was 214 bp.

### Combined bisulfite restriction analysis (COBRA)

Bsh1236I restriction enzyme was used to digest the amplified region of the GHR gene (214 bp) using the Thermo Scientific, Fast-Digest Kit. The total reaction volume was 15 µL containing 1 µL of the (Bsh1236I) enzyme, 1 µL of fast digest green buffer, 5 µl PCR products, and 8 µL water nuclease-free. The mixture was mixed well and incubated for 5 min at 37 °C, then loaded onto 6% polyacrylamide gel electrophoresis to detect the digested bands (124 bp, 63 bp, and 27 bp).

### RNA extraction and reverse transcriptase-PCR

Total RNA was extracted by the RNA isolation kit (Qiagen, GmbH, Germany) according to the manufacturer’s protocol. Then, RT-PCR was carried out using the RT-PCR Kit (Qiagen, GmbH, Germany), where 5 μg ~ 11μL of total RNA was used. The PCR profile started with incubating the reaction mixture at 65 °C for 5 min followed by a second incubation after adding 4 μL of 5 × reaction buffer, 1 μL of Ribo-Lock RNase inhibitor (U/μL), 2 μL of 10 mM dNTP mix, and 1 μL of Revert-Aid M-MulV RT (200 U/μL) up to 20 µL as a total reaction volume at 42 °C for 60 min, and inactivation at 70 °C for 5 min.

### Quantitative real-time PCR (qRT-PCR)

The generated cDNA has been subjected to Real-Time PCR using primer sets for GHR, GDF-9 genes, and RPLSP0 as a housekeeping gene (Table 2), according to Thermo Scientific-Maxima SYBR green qPCR master mix (2x), using ROTOR-GENE machine, (Qiagen GmbH, Germany). The total reaction volume was 25 µL containing 12.5 µL of SYBR green master mix (2x), 1.5 µL (10 pmol) from each primer, 2.5µL from cDNA and nuclease-free water up to 25 µl. The thermal-cycler program started with an initial denaturation at 95 °C for 15 min. and 40 cycles of 95 °C for 15 s. for denaturation, 60 °C for 30 s. for annealing, and 72 °C for 30 s. for extension, and final extension at 72 °C for 5 min. For reproducible results, the PCR reaction for each gene was assessed in triplicates. The target mRNA amount was determined and normalized relative to the amount of Ribosomal Protein Large Subunit P0 (RPLSP0) mRNA. For calculating fold change expression relative to the reference gene (RPLSP0), the formula (2 − ^ΔΔCt^) was estimated.

**Table 2:** Real-time primer sets

QRT-PCR Primers of the GDF-9 Gene
Forward: 5′- GACGCCACCTCTACAACACT-3′
Reverse: 5′- ACGATCCAGGTTAAACAGCAGA-3′
QRT-PCR Primers of the GHR Gene
Forward: 5′- CATAGTGCGGTCTGCTTCCA-3′
Reverse: 5′- GTGTGGCTTCACTCCCAGAA-3′
Ribosomal Protein Large Subunit P0 Reference (RPLSP0) Gene For QRT PCR
Forward: 5′- CAACCCCGAAGTGCTTGACAT-3′
Reverse: 5′- ACGCAGATGGATCAGCCA-3′

### Statistical analysis

Analysis of variance was reach tested by the general linear model (GLM) procedure of the statistical analysis system (SAS 2004) was used to test the effect of the studied fixed factors (season, level of production, litter size, parity, GDF-9 /Methylation (MSP), and GHR/Restriction enzyme) on the peak milk yield and total milk yield traits and the assumed models were:

5 $$Y_{ijklmnn \, = } \mu + \, S_{i} + \, P_{j} + \, L_{k} + \, T_{l} + \, M_{m} + \, R_{n} + \, e_{ijklmnn}$$

where,

**Y**_**ijklmnn**_ is the observed records of peak milk yield of the n^th^ doe (after 14 days of birth) and the total milk yield of the n^th^ doe (per season);

**µ** is the overall population means;

**S**_**i**_ is the fixed effect of ith season of birth, i = 1: (November) and i = 2: (March);

**P**_**j**_ is the fixed effect of jth production levels, j = 1: (High) j =2: (Low);

**L**_**k**_ is the fixed effect of kth litter size of doe, k = 1: (Single) k = 2: (multiple birth);

**T**_**l**_ is the fixed effect of lth parity of does, l = 3 to 5;

**M**_**m**_ is the fixed effect of mth methylation of GDF-9 gene, m = 1: (methylated), m = 2: (unmethylated), and m = 3: (hemimethylated);

**R**_**n**_ is the fixed effect of n^th^ methylation of GHR, n = 1: (unmethylated, not digested), n = 2: (hemimethylated, digested to two bands), and n = 3: (methylated, digested to three bands);

**e**_**ijklmnn**_ is the random residual associated with the individual, assumed to be independent and normally distributed with (0, ${\sigma }_{e}^{2}$).

$$Y_{imnn} = \mu \, + \,S_{i} \, + \,M_{m} \, + \,R_{n} \, + \,e_{imnn}$$

where,

**Y**_**imnn**_ is the observed records of the nth doe of gene expression of GDF-9 and GHR genes;

**µ** is the overall population means;

**S**_**i**_ is the fixed effect of ith season of birth, i = 1: (November) and i = 2: (March);

**M**_**m**_ is the fixed effect of mth methylation of GDF-9 gene, m = 1: (methylated), m = 2: (unmethylated), and m = 3: (hemimethylated);

**R**_**n**_ is the fixed effect of nth methylation of GHR, n = 1: (unmethylated, not digested), n = 2: (hemimethylated, digested to two bands), and n = 3: (methylated, digested to three bands);

**e**_**imnn**_ is the random error.

The effect of PMY on TMY and simple regression models were:

$${\mathbf{Y}}_{{\mathbf{q}}} = \, {{\varvec{\upmu}}} \, + \, {\mathbf{X}}_{{\mathbf{q}}} + \, {\mathbf{e}}_{{{\mathbf{qn}}}}$$

where,

**µ** is the overall population means;

**X**_**q**_ is the fixed effect of q^th^ peak milk yield, q = 1: (6 ≤ 30 kg), 2: (30 ≤ 40 kg), 3: (40 ≤ 50 kg) and 4: (> 50 kg);

**e**_**qn**_ is the random error.

$${\mathbf{Y}} \, = \, {\mathbf{a}} \, + \, {\mathbf{bx}} \, + \, {\mathbf{e}}\,\left( {\text{Simple Linear Regression}} \right)$$

where,

**Y** is the total milk yield of the nth doe (per season);

**a** is the Intercept, and it is the value when x = 0;

**Bx b** is the co-efficient of regression, and **x** is the fixed effect of peak milk yield (after 14 days of birth);

**e** is the random error and it is assumed to be independent and normally distributed with (0, ${\sigma }_{e}^{2}$).

## Results

### Differences between high and low milk production on PMY and TMY

Milk samples were collected from one hundred mature female Zaraibi dairy goats divided into two groups: the high milk yield group which produces more than 202.17 kg milk per season (about 46 does) and the low milk yield group which produces less than 202 kg milk per season (about 54 does) according to total milk yield. Differences between high and low milk production were highly significant (*P* ≤ 0.001) in both the peak milk yield and total milk yield (Table 3).

**Table 3:** Differences between high and low milk production on PMY and TMY

Factors	Category	N	PMY(kg)	TMY(kg)
			M ± SE	M ± SE
Milk Production-level	High	46	46.0^a^ ± 2.43	259.5^a^ ± 9.6
Overall mean	Low	54	32.4^b^ ± 3.00	153.3^b^ ± 9.4
		100	38.63 ± 1.08	202.17 ± 3.25

*mean (M) ± standard error (SE); a, b, c = significantly different at *P* < 0.05; peak milk yield (PMY), total milk yield (TMY) and N: number of records

### Effect of peak milk yield on TMY

**Fig. 1:**
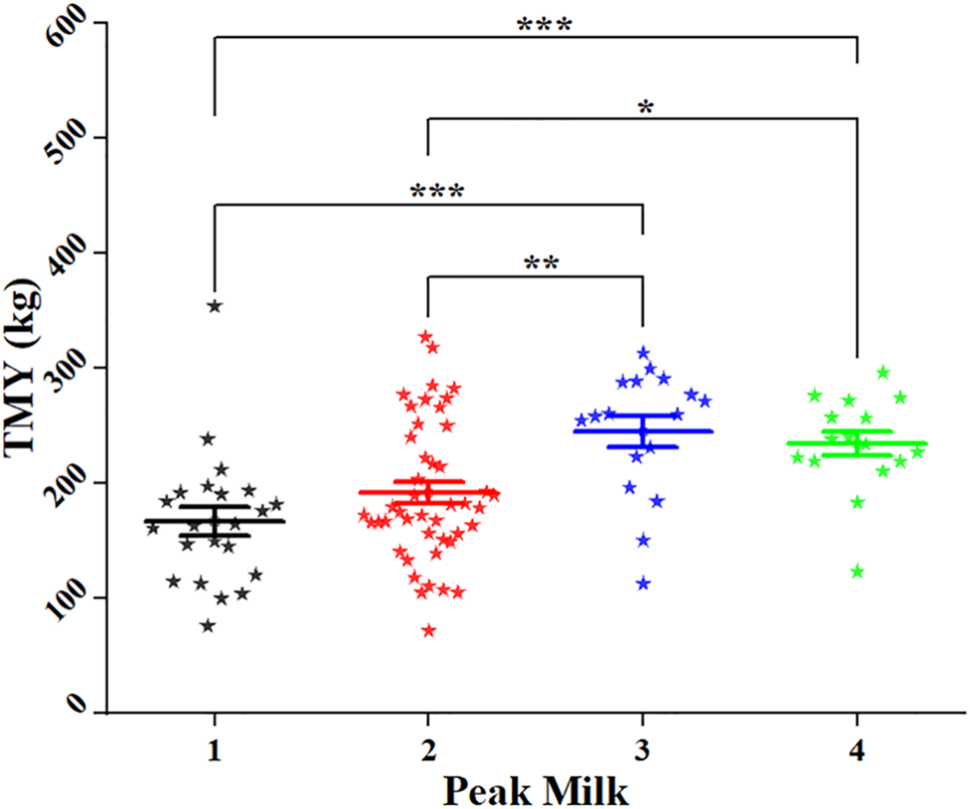
Effect of peak milk yield (PMY) through 14:21 days on total milk yield (TMY) (/season); a, b, c = is significantly different at *P* < 0.05, PMY categories (based on the milk yield/kg.) = 1: (6 ≤ 30 kg), 2: (30 ≤ 40 kg), 3: (40 ≤ 50 kg) and 4: (> 50 kg).* = significance *P* ≤ 0.05, *P* ≤ 0.01, ** = significance *P* ≤ 0.03, *** = highly significant, non-significant *P* > 0.05 (no*)

The effect of PMY through 14:21 days on TMY (/season) was highly significant between the 1, 2, 3, and 4 categories based on the milk yield/kg, and means (M) ± standard error (SE) (167.1^b^ ± 12.1, 191.7^b^ ± 8.4, 244.7^a^ ± 13.7, and 234.5^a^ ± 14.2, respectively), but non-significant between categories 1 and 2, and between categories 3 and 4 (Fig. 1).

The regression correlation between PMY on TMY was highly significant (*P* ≤ 0.0001), showing a positive effect, when PMY increased by 1 kg, the TMY would be increased by 1.45 kg (Table 4).

**Table 4:** Regression correlation between peak milk yield and total milk yield

Variable	D.F	Parameter estimate	*Pr* >|t|
Intercept	1	146.12	< 0.0001
PMY	1	1.45 kg	0.0005

Results were expressed as simple linear regression when Y (TMY) = 146.12 + 1.45x.

### Sequencing analysis of bisulfite-converted DNA

**Fig. 2:**
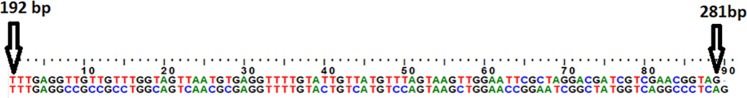
Alignment of the genomic DNA of Zaraibi goat and its converted DNA showing the conversion of each unmethylated nucleotide “C” to nucleotide "T"

Conversion of genomic DNA was proven by sequencing analysis of part of the genomic DNA from nucleotide number “192” to nucleotide number “281” in the promoter sequence of GDF-9 gene where each unmethylated "Cytosine" nucleotide converted to “Thymine” (Fig. 2).

### Prediction of the CpG island and methylation primer design

**Fig. 3:**
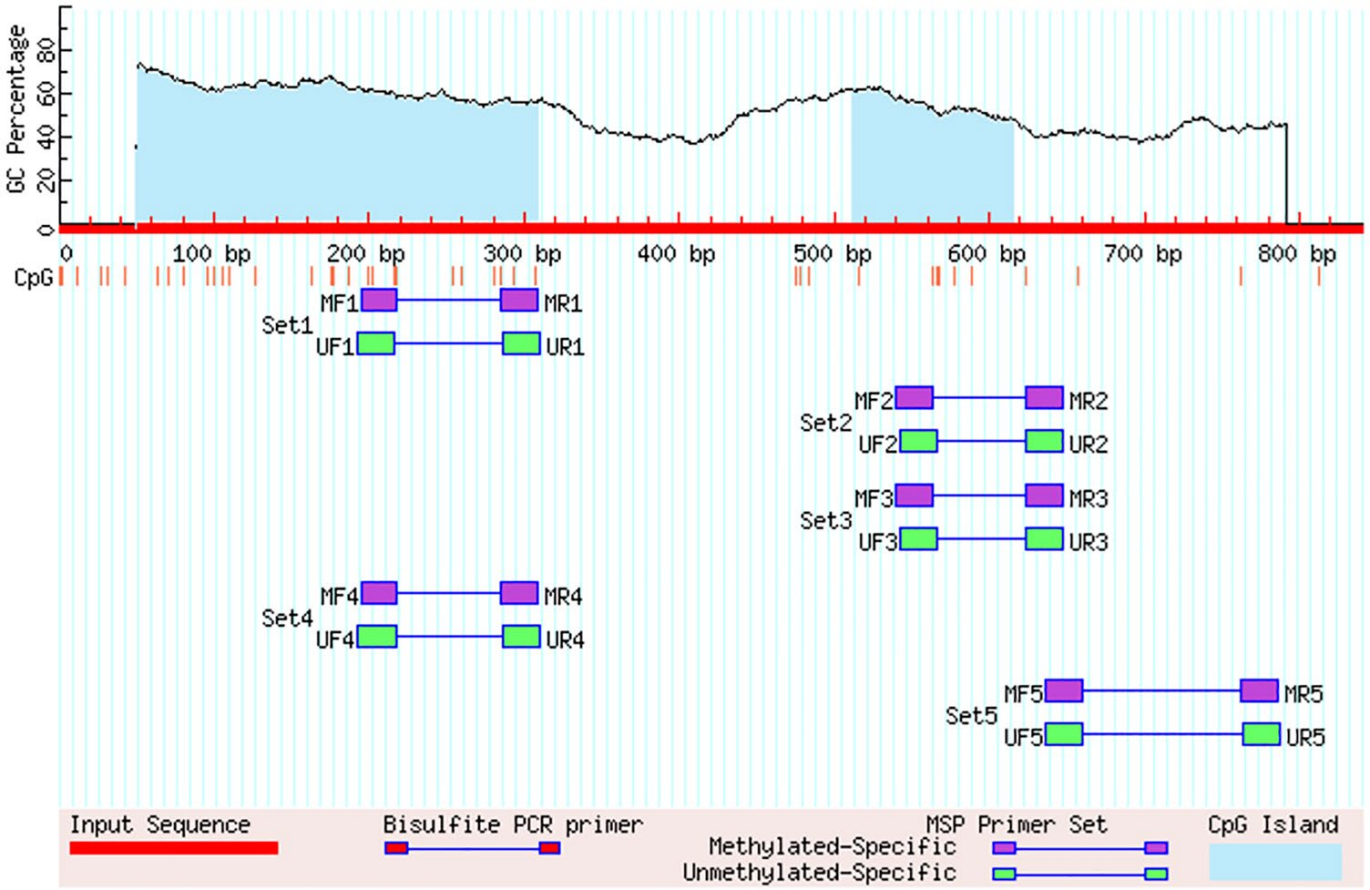
The promoter region of the GDF-9 gene shows the two detected CpG islands

**Fig. 4:**
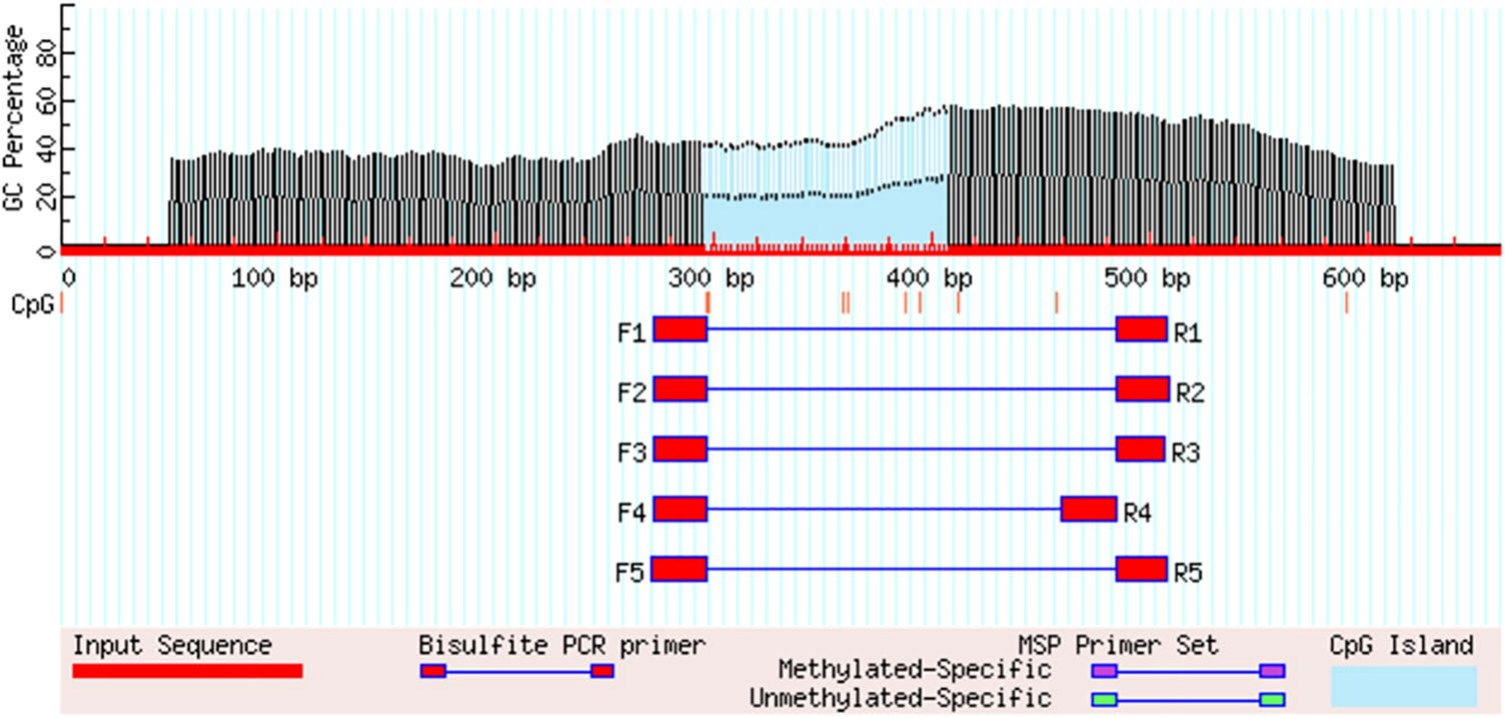
The promoter region of the GHR gene shows one detected CpG islands

A free online tool Meth-Primer https://www.urogene.org/methprimer/ was used to identify CpG islands within the promoters of GHR and GDF-9 genes and to design primers used to amplify these CpGs specific regions (Li and Dahiya 2002).The sequence length of GDF-9 was 840 bp where two CpGs islands in the promoter region of the GDF-9 gene were found, island (1) size was 261 bp (from 49 to 309 bp), island (2) size was 104 bp (from 512 to 615 bp) (Fig. 3). Otherwise, GHR gene had a 660 bp of sequence length and one CpG island in the promoter region was found, the size island (1) was 113 bp (from 295 to 407 bp) (Fig. 4).

### DNA methylation pattern of the GDF-9 gene promoter using MSP analysis

**Fig. 5:**
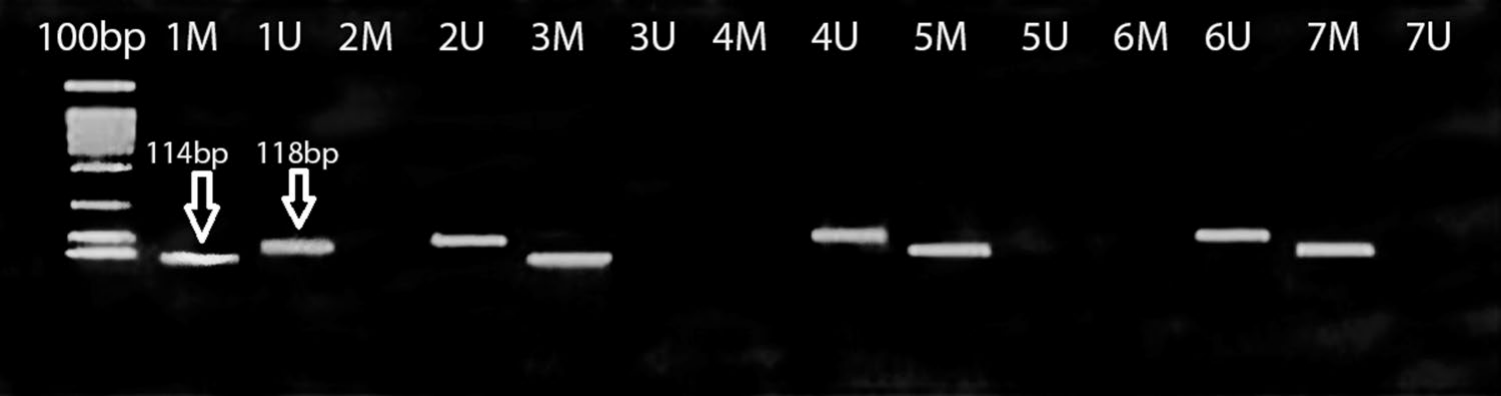
PCR products of MSP for the GDF-9 gene, lane 1: DNA ladders at 100 bp. M: methylated PCR products at 114 bp. U: Unmethylated products at 118 bp for samples 1–7. (1 M and 1U): hemimethylated sample showing methylated and unmethylated PCR products at114 bp and 118 bp, respectively

The methylated samples showed a successful PCR product of CpG1 at 114 bp, while for the un-methylated samples; a sharp band at 118 bp was reported. Some samples revealed two bands in the same samples (PCR of methylated and unmethylated pattern) that were called hemimethylated samples, as indicated in Fig. 5. While CpG 2 region showed no PCR products after using two different sets of methylated and unmethylated primers (set 2 and set 3 Fig. 3).

### Percentage of methylation levels of the GDF-9 gene in high and low milk production Zaraibi Goat

**Fig. 6:**
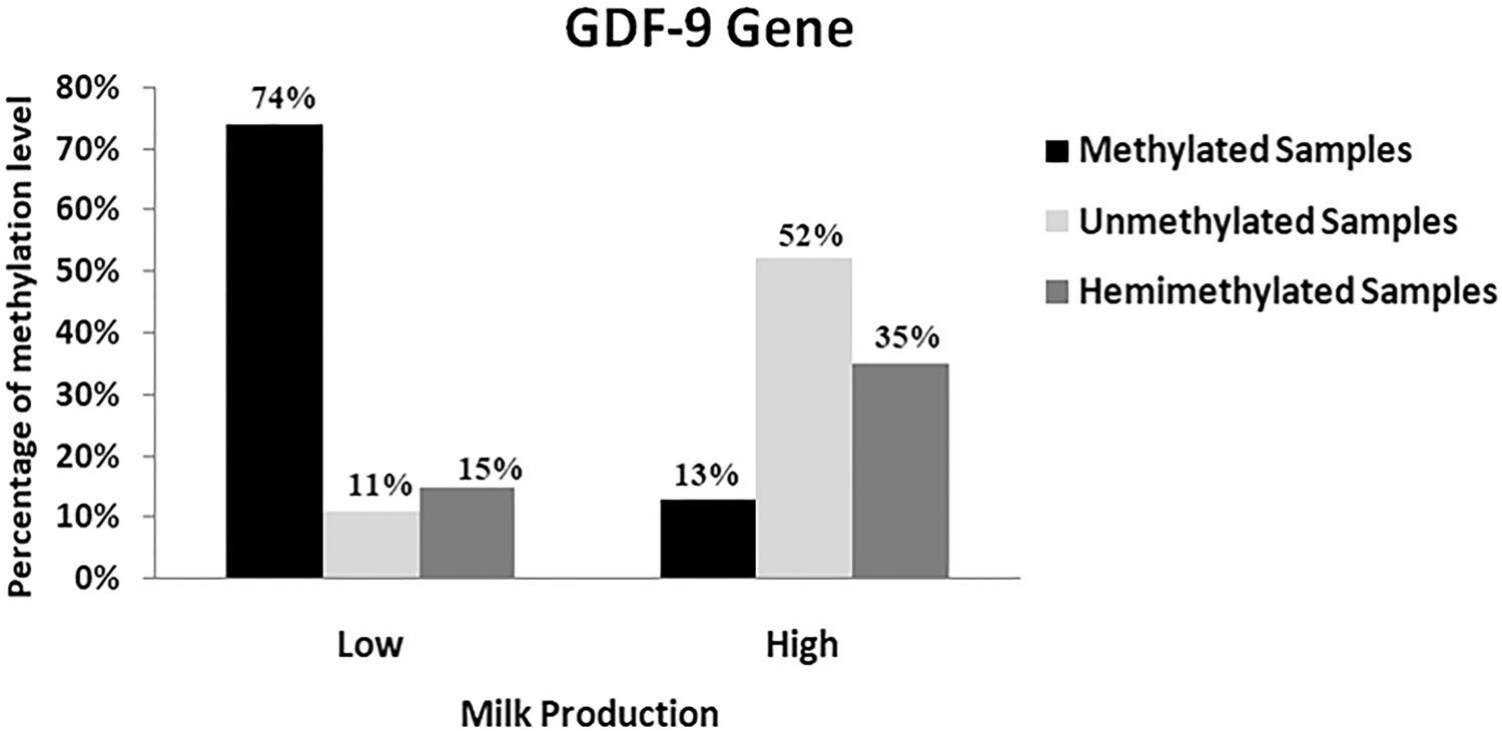
Percentage of methylation levels of the GDF-9 gene within high and low milk production

From 54 samples of low milk production samples, there were 74% methylated, 11% unmethylated, and 15%, hemimethylated samples. On the other hand, the percentages of unmethylated, methylated, and hemimethylated samples in the total number of high milk production samples were 52%, 13%, and 35%, respectively out of 46 samples (Fig. 6).

### DNA methylation pattern of GHR gene promoter using COBRA analysis

**Fig. 7:**
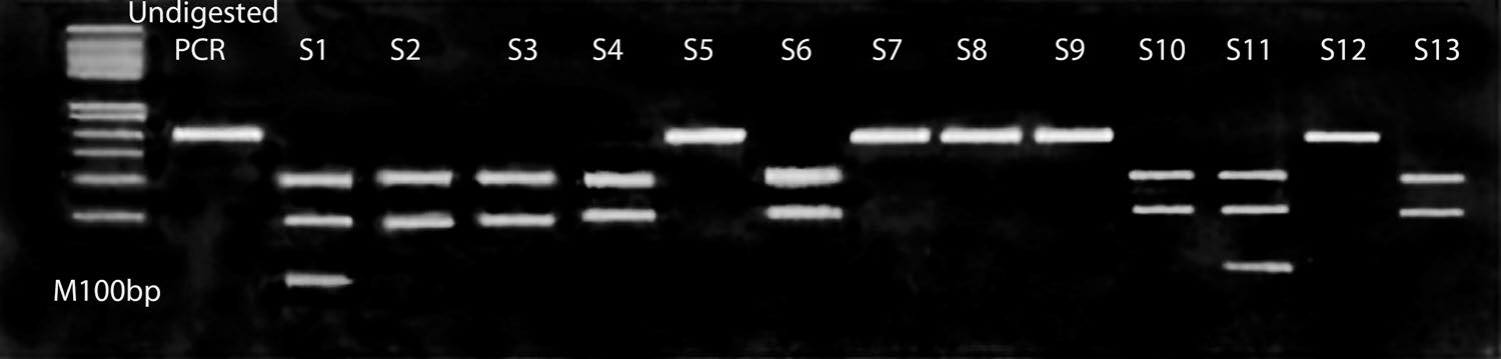
Bands of GHR PCR products after digestion with restriction enzyme (Bsh1236I). Lane 1: DNA ladders at 50 bp, lane 2: undigested sample. Lanes (s1, s11): methylated samples digested to three bands 124 bp, 63 bp, and 27 bp. Lanes (s2, s3,s4, s6, s10, s13): hemimethylated samples digested to two bands 124 bp and 63 bp. Lanes (s5, s7, s8, s9, s12): unmethylated samples showed only one band at 214 bp

The digestion of the GHR amplicon (214 bp) using the enzyme (Bsh126I) showed three bands at 124 bp, 63 bp, and 27 bp at complete digestion (Methylated samples), and only two bands at 124 bp, 63 bp for hemimethylated samples, and a sharp band at 214 bp when the amplicon was not digested (Unmethylated samples) (Fig. 7).

### Percentage of methylation levels of GHR gene in high and low milk production

**Fig. 8:**
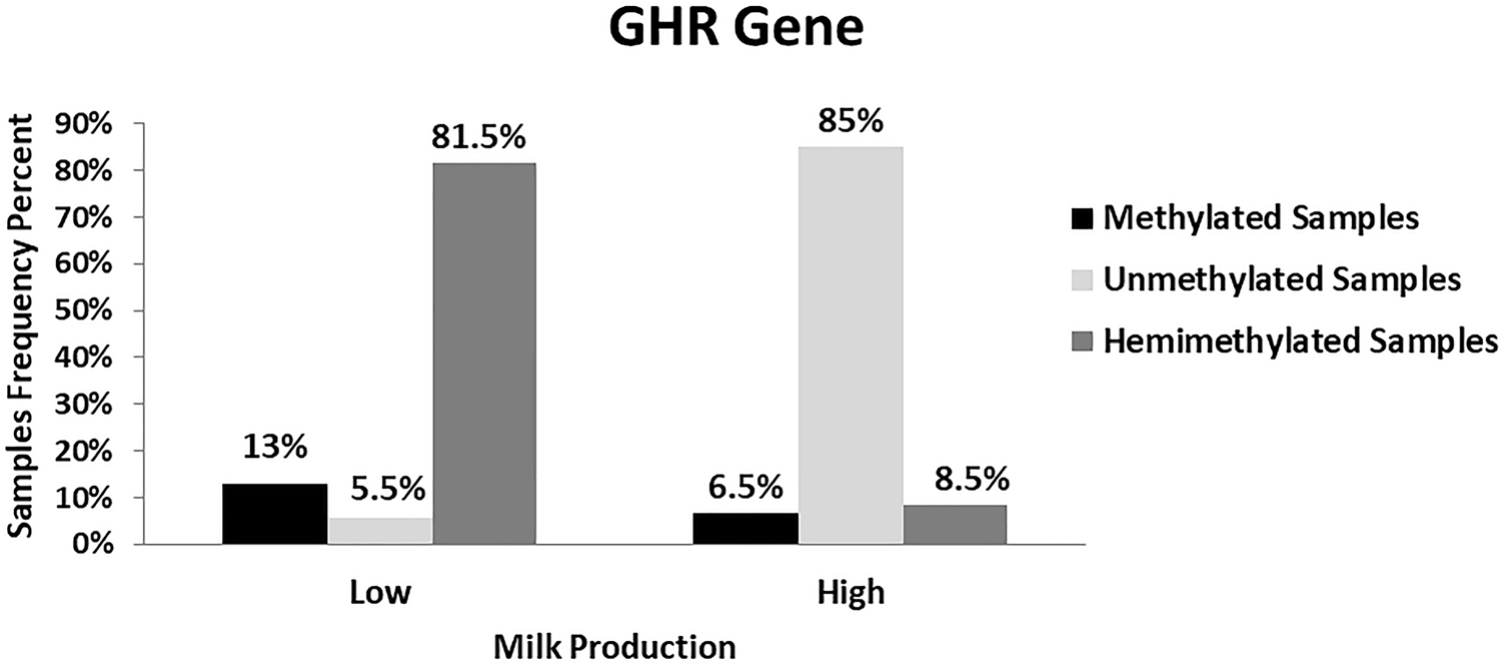
Percentage of GHR gene methylation levels within high and low milk production

Approximately 81.5%, 13% and 5.5% of 54 samples of low milk production samples were hemimethylated samples (digested into two bands), methylated samples (digested into three bands), and unmethylated samples, respectively. While in 46 high milk production samples, there were 85% unmethylated samples, 8.5% hemimethylated samples and 6.5% methylated samples (Fig. 8).

### mRNA expression levels of GDF-9 and GHR genes

**Fig. 9:**
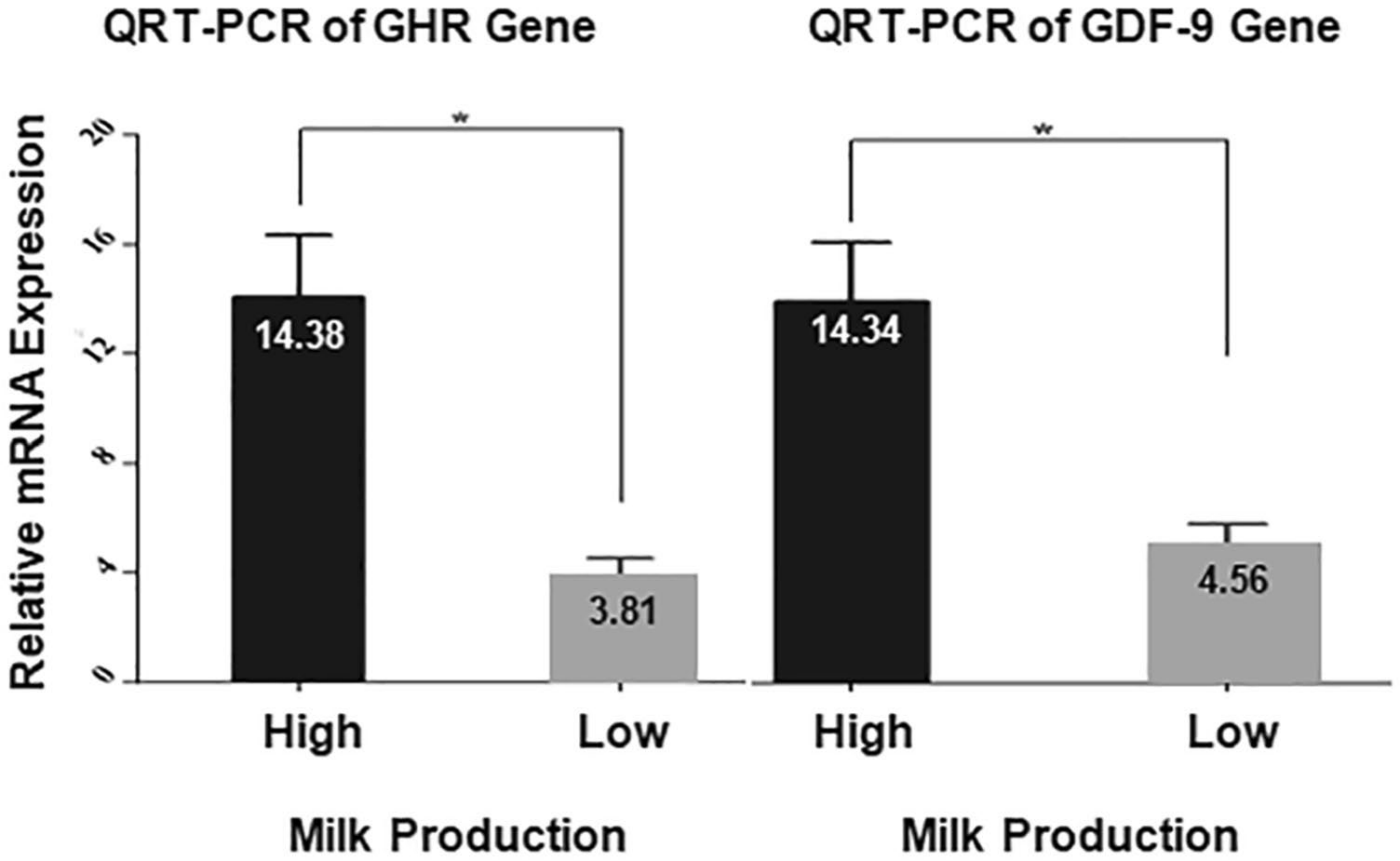
Relative mRNA expression of GHR and GDF-9 genes in milk production groups. Data expressed relative to the housekeeping gene RPLSP0. The data represent the mean ± SE, (n = 100). * = indicated the significant difference (*P* < 0.05)

Quantitative real-time PCR was used to analyze the mRNA levels of *GDF-9 and GHR* gene expression in high and low milk production groups. The expression levels of both GDF-9 and GHR genes in high milk yield samples were significantly *(P* < 0.05) higher than the expression level of low milk yield samples (Fig. 9).

### Relationship between methylation patterns and expression level of GDF-9 and GHR genes

**Fig. 10:**
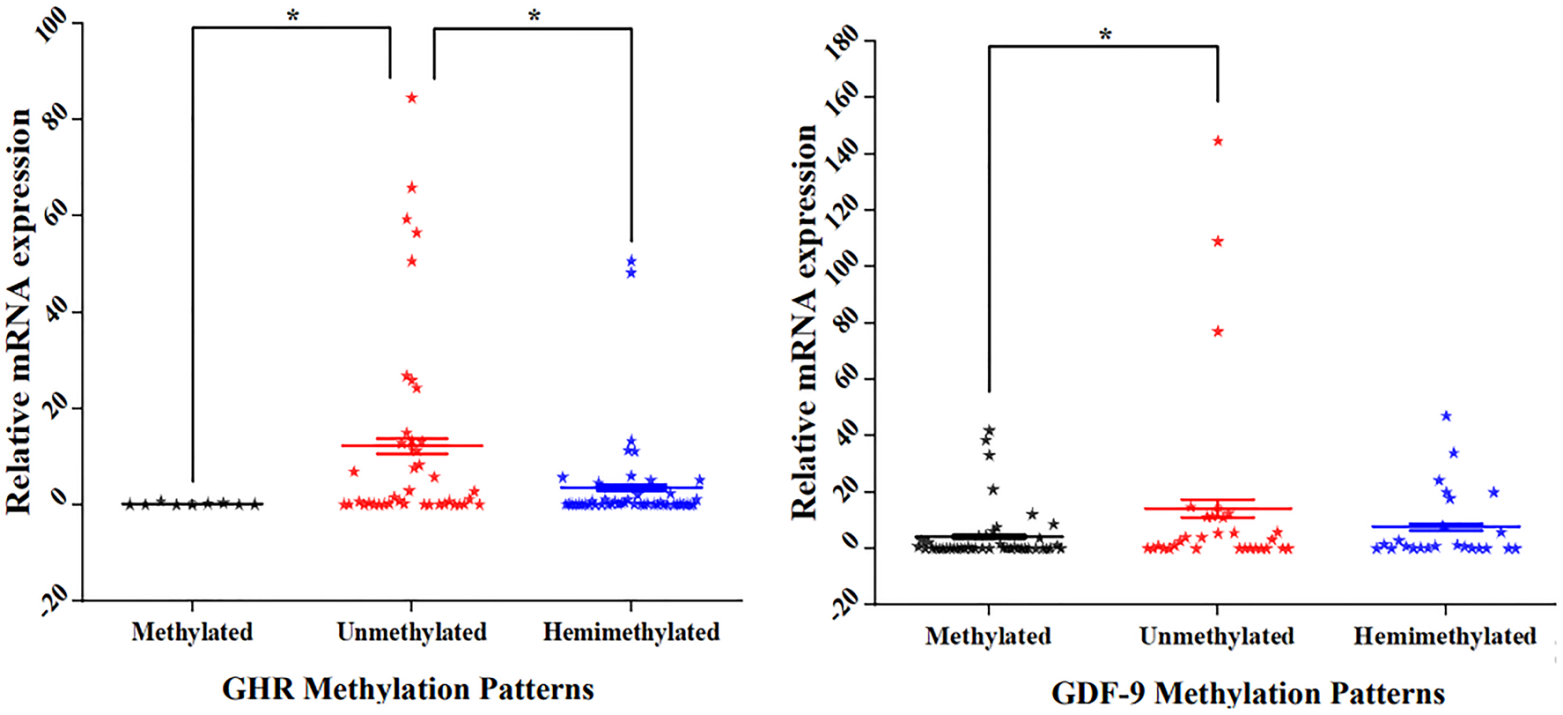
The effect of methylation patterns of GHR and GDF-9 genes on their relative mRNA expressions. The data represent the mean ± SE, (n = 100). * = indicated the significant difference (*P* < 0.05)

mRNA expression of the GHR and GDF-9 genes showed a significant effect (*P* < 0.05) between methylated and unmethylated samples (Fig. 10).

### Relationship between breeding season and the expression levels of GDF-9 and GHR genes

**Fig. 11:**
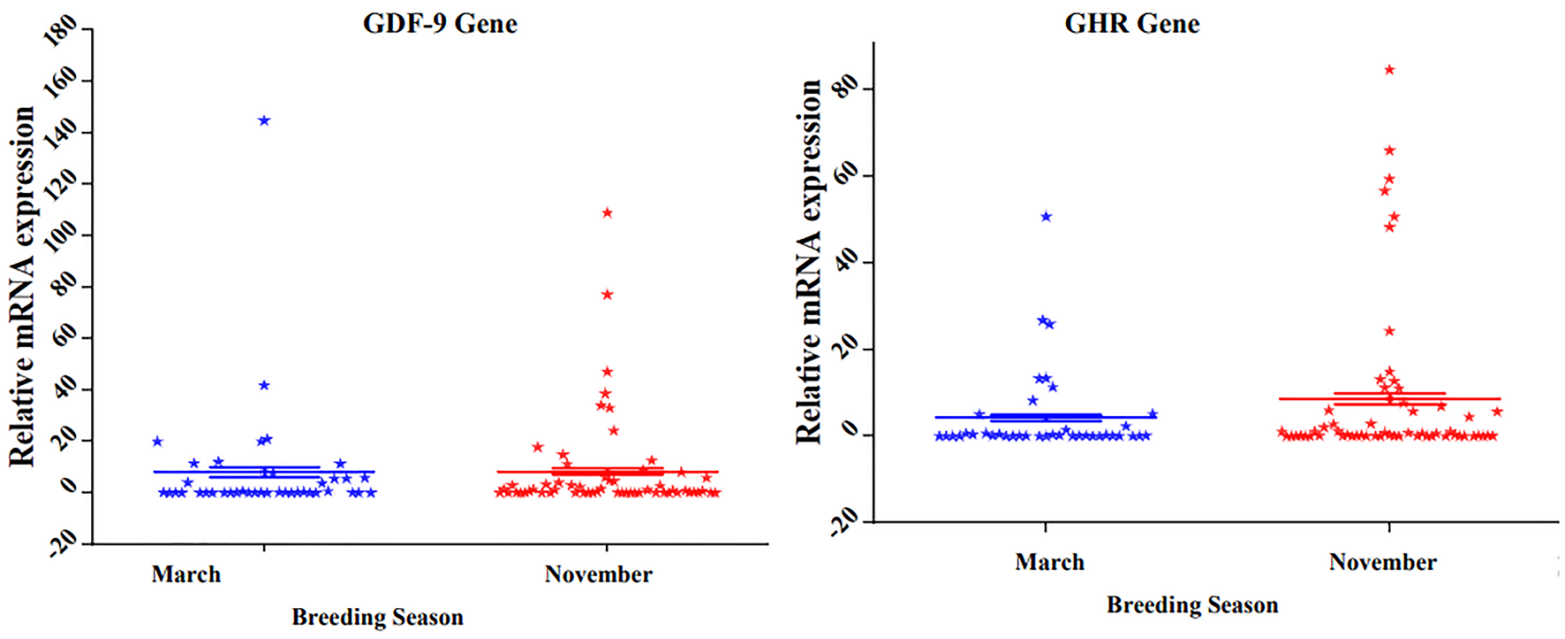
Effect of breeding season on the expression of GHR and GDF-9 genes. The data represent the mean ± SE, (n = 100)

Breeding season showed a non-significant effect *(P* > 0.05) in (March and November) on the expression of both genes (GDF-9 and GHR) (Fig. 11).

### Effect of methylation levels of GDF-9 and GHR genes on PMY & TMY

The effect of the methylation level of GDF-9 and GHR genes on both peak milk yield (PMY) and total milk yield (TMY) were highly significant (*P* < 0.05) in unmethylated, methylated, and hemimethylated patterns (48.0^a^ ± 2.3, 30.2^b^ ± 2.0, and 43.0^a^ ± 2.6 kg, respectively) on PMY, while they were (230.0^a^ ± 7.6, 170.5^b^ ± 6.5, and 228.3^a^ ± 7.7 kg, respectively) on TMY of the GDF-9 gene. Among the three categories, there was a highly significant difference (*P* < 0.05) between methylated and unmethylated patterns and between methylated and hemimethylated patterns on PMY and TMY, but there is no discernible difference among unmethylated and hemimethylated levels on PMY and TMY. Regarding the methylation pattern of GHR gene, there was a highly significant difference (*P* < 0.05) between unmethylated pattern, hemimethylated pattern, and methylated pattern (47.8^a^ ± 2.4, 33.0^b^ ± 2.4, and 26.3^c^ ± 3.6 kg, respectively) on PMY and (252.7^a^ ± 7.4, 195.9^b^ ± 11.0 and 159.2^c^ ± 7.3 kg, respectively) on TMY. Moreover, there was a hugely significant difference (*P* < 0.05) in the methylated pattern more than both unmethylated and hemimethylated patterns of GDF-9 on PMY and TMY, whereas, the three patterns of GHR showed a remarkably significant difference *(P* < 0.05) between them on PMY and TMY (Table 5).

**Table 5:** Effect of methylation of GDF-9 and GHR genes on PMY and TMY

Factors	Methylation patterns	N	PMY(kg)	TMY(kg)
			M ± SE	M ± SE
Methylation-GDF-9	Unmethylated	30	48.0^a^ ± 2.3	230.0^a^ ± 7.6
	Methylated	46	30.2^b^ ± 2.0	170.5^b^ ± 6.5
	Hemimethylated	24	43.0a ± 2.6	228.0^a^ ± 7.7
Methylation-GHR	Unmethylated	42	47.8^a^ ± 2.4	252.7^a^ ± 7.4
	Methylated	10	26.3^c^ ± 3.6	159.2^c^ ± 7.3
	Hemimethylated	48	33.0^b^ ± 2.4	195.9^b^ ± 11.0

*mean (M) ± standard error (SE); a, b, c = different letters are significantly different at *P* < 0.05; peak milk yield (PMY), total milk yield (TMY) and N: number of records

### Effect of some environmental factors (breeding season, litter size, and parity) on peak milk yield (PMY) and total milk yield (TMY)

Breeding season showed a higher significant difference *(P* < 0.05) in the March season than November one on PMY (42.5^a^ ± 1.7 and 32.7^b^ ± 2.2, respectively), meanwhile, there wasn’t a significant difference between both seasons on TMY. On the other hand, single litter size was highly significant (*P* < 0.05) on TMY than PMY (229.5^a^ ± 6.1 and 187.4^b^ ± 5.8, respectively), while litter size showed no significant difference between them on PMY. Regarding the parity (kidding season), there were highly significant differences *(P* < 0.05) between the 3rd, 4th, and 5th parities on TMY (204.5^b^ ± 8.3, 260.2^a^ ± 8.1, and 157.4^c^ ± 8.0, respectively), while PMY at 3rd and 4th parities weren’t significantly different, but there was a discernible difference among 3rd and 4th comparable to the 5th (41.3^a^ ± 2.8, 41.9^a^ ± 2.7 and 34.7^b^ ± 2.7, respectively). Taken together, the 3rd and 4th parities reported the highest significant difference (*P* < 0.05) in PMY, while the 4th parity was the most effective one on TMY (Table 6). Collectively, all the tested factors were significantly effective on PMY and TMY, where the 4th parity showed the highest effect on TMY.

**Table 6:** Effect of the breeding season, litter size, and parity on peak milk yield (PMY) and total milk yield (TMY)

Factors	Category	N	PMY(kg)	TMY(kg)
			M ± SE	M ± SE
Breeding Season	March	60	42.5^a^ ± 1.7	205.1^a^ ± 5.1
	November	40	32.7^b^ ± 2.2	197.7^a^ ± 6.6
Litter Size	Single	35	38.8^a^ ± 2.0	229.5^a^ ± 6.1
	Multiple	65	38.5^a^ ± 1.9	187.4^b^ ± 5.8
Parity	3rd	23	41.3^a^ ± 2.8	204.5^b^ ± 8.3
	4th	33	41.9^a^ ± 2.7	260.2^a^ ± 8.1
	5th	44	34.7^b^ ± 2.7	157.4^c^ ± 8.0

*mean (M) ± standard error (SE); a, b, c = different letters are significantly different at *P* < 0.05; peak milk yield (PMY), total milk yield (*TMY*), and *N* number of records

## Discussion

DNA methylation is considered a regulatory tool for mammary gland development (Chen et al. 2019). A recent study has reported a potential association between DNA methylation and milk production (Wang et al. 2021). GHR and GDF-9 genes play a crucial role during lactation. GHR gene and its polymorphisms have a vital role in the development of the mammary gland and milk production (El-Komy et al. 2020; Nanaei et al. 2020; Cobanoglu et al. 2021; Erdoğan et al. 2021). Several studies indicated that GDF-9 increases ovarian follicles growth, and ovulation rate, and its polymorphisms had a significant correlation with litter size, number of lambs, twining %, and thus milk production (Al-Khuzai and Ahmed 2019; Koyun, et al. 2021). Therefore, it is meaningful to study the methylation status of these genes and their association with milk production performance. The present data revealed that DNA methylation patterns of the GDF-9 and GHR have high significant differences (*P* < 0.05) in PMY and TMY, in high and low milk production groups. There was a positive correlation between the quantitative mRNA expression of GDF-9 and GHR with milk production, and a negative correlation between their methylation percentage and milk production, where high methylation states of GDF-9 and GHR genes were associated with the reduction of milk production in Zaraibi goat. This may be because when CpG islands in the promoter regions are methylated abnormally, remodeling of chromatin conformation takes place and gene transcription is suppressed (Cedar and Bergman 2009, Li and Zhang 2014). This is similar to the results of (Wang et al. 2019a, b) who reported that high milk yield of dairy cows was associated with low methylation percentage, while those of low milk yield have higher methylation percentage. Also this may contribute to the down-regulation of DNMT3A and 3B in high milk yield animals, and miR-29 s in the low milk yield group as revealed by (Bian et al. 2015) in dairy cows, where miR-29 s inhibits the expression of De novo methyltransferase enzymes DNMT3A and 3B,which indicates the role of DNA methylation as a regulatory mechanism of mammary function. Some recent studies showed that the production of milk in cows is a complicated feature that is influenced by several biological and environmental variables. According to Wang et al*.* (2019), in high-milk yield cows, DNA methylation rates were found to be lower. Xuan Liu et al*.* (2017), discovered that DNA methylation of EEF1D gene may have a significant impact on milk production traits in dairy cattle and likely plays a significant role in its transcriptional regulation. Wanting et al*.* (2020), compared the transcriptional profiles and genome-wide DNA methylation patterns of cows with highly diverse milk production performances using genome-wide DNA methylation sequencing and RNA-seq on blood tissue, and revealed that blood tissue alterations in DNA methylation and gene expression for the DOCK1, PTK2, and PIK3R1 genes had variations in milk production among cattle. In addition, the quantity of methionine, lysine, choline, and folate in the diet, among other dietary elements that impact milk supply and composition, has been shown by Ana Lesta et al. (2023) to change the methylation status of certain genes in dairy cows. According to Jiang et al*.* (2014), the EEFID gene and the ribosome 60S were both shown to be highly expressed in the mammary tissue during the milking stage in cows. According to Xiaoyun et al*.* (2023), photoperiod may cause the DNA of the MTNR1A gene to be methylated to regulate the gene's expression. The levels of DNA methylation and gene expression in ewes had a substantial negative connection (*P* < 0.001) that changed how reproductive hormones were secreted and influenced the sheep's seasonal reproductive activity. However, the present study was not designed to measure the expressions of such genes. We can indicate that DNA methylation plays a significant role in milk production as stated by (Singh et al. 2010; Hwang et al. 2017).

The effects of non-genetic factors on PMY and TMY were remarkably significant within the high and low milk yield groups. Breeding season was profoundly significant (*P* ≤ 0.001) on both, PMY and TMY, there were no significant differences between March and November seasons on TMY, while the March season showed a higher significant difference on PMY than November. This agrees with (Akpa et al. 2001) who discovered that does with kidding during the wet season (November through February) had a lower PMY of (2.16) kg than those with kidding during the dry season (March through October) who had PMY of (2.34 kg). A similar study demonstrated that lower milk production has been reported in winter at the beginning of lactation, than those kidding in the spring (León et al. 2012; Arnal et al. 2018). That may be rationalized by the availability of food in terms of quality and quantity during dry seasons including crop residues and grazable materials which means there is no nutritional stress. The relationship between food quality and milk production has been reported by (McCarthy et al. 2011; Hanrahan et al. 2018; Hennessy et al. 2020) who stated that one cow showed increasing in stocking rate per hectare resulted in increasing of milk production by 20% per hectare, which requires a reconsideration of grassland organization for pasture use to increase productivity on the farm. Moreover, March was reported as the lowest CMY and shortest lactation length in dairy goats in Australia due to the short photoperiod according to (Zamuner et al. 2020), that is the case during wet seasons in Egypt (November through February) where they show short photoperiod and thus milk production is reduced. Regarding the effect of litter size of does on PMY, it was non-significant (*P* ≥ 0.05) in both single and multiple births, but highly significant on TMY in a single birth. These findings are similar to (Wahome et al. 1994) who found that the litter size was not significant in an increase of peak milk yield and the decline after peak yield, but it was significant (*P* < 0.01) in total milk yield in dairy goats. In addition, (Akpa et al. 2001), reported a non-significant effect on litter size for both PMY and TMY in Red Sokoto goats in single and twins birth. Considering that all the previous reports were taken from true ranges conditions; these findings may be related to the weak conditions resulting from the stress of pregnancy and birth of twins or triplets. However, these observations were controversial with (Margatho et al. 2019; Zamuner et al. 2020) who reported that CMY was higher in animals delivering multiple kids than those delivering a single kid. The high proportion of alveoli along several lactation periods increases the udder volume and the secretory parenchyma, and thus increases the milk production in multiparous goats compared with primiparous goats. In this study, the effect of parity on milk productivity varied as goats go through lactation, where Zaraibi had the lowest milk yield at the first parity, while 3rd, 4th, and 5th parities showed a highly significant effect (*P* ≤ 0.01) on PMY and TMY. These results are supported by several authors who stated that in many goat breeds (Zaraibi, Baladi, Damascus, Angola-Nubian, and Angora), the maximum milk yield was achieved at 3rd, 4th and 5th parities (Teleb et al. 2009; Hamed 2010; Anwar et al. 2012). Conversely, a recent report stated that the fourth parity in goats showed the shortest lactation period, while the third parity was reported as the period of maximum milk production (Zamuner, et al. 2020). Similarly, a peak with larger perseverance in first-parity goats, and reduced perseverance with rising parity was observed by (León et al. 2012; Arnal et al. 2018). The present study showed that the mRNA expression of both genes (GDF-9 and GHR) was not significantly different throughout the breeding season (*P* > 0.05) in either of the two seasons (March or November). By comparing the effect of methylation patterns on the mRNA expression of genes (GDF-9 and GHR) it was revealed that there was significant e effect (*P* ≤ 0.05) between methylation, unmethylated and hemimethylated patterns and low, high and medium milk production which indicated that methylation changes had direct significant effects when compared with breeding season and their effects on milk production. These results agree with Sushil et al*.* (2020), who showed that there was no significant effect of season on different productive performance traits in Sahiwal cattle. Although, Xuan Liu et al*.* (2017) revealed that the methylation changes in the dry period was less than at the early stage of lactation, and the mRNA expression of EEF1D was greater in the dry period than it was at the early stage of lactation. So, that could demonstrate the relationship between methylation patterns, gene expression and their association with milk production. Regression co-relational of PMY (14:21 day after birth) with TMY (240 days) was highly significant (*P* ≤ 0.001). It indicated a positive co-relational statistic, where an increase of PMY by 1 kg causes an increase of TMY by 1.45 kg. These results agree with (Abdelhamid et al*.* 2011) who estimated that a total milk yield of 363.15 kg in Zaraibi does for 240 days, tended to decrease during the suckling and lactation period in low yield does more than high yield animals.

This is the first study that gives additional information on how methylation pattern of GHR and GDF-9 may affect the milk production of commercial dairy goats, and also has produced new knowledge regarding the effects of different non-genetic factors on milk yield in Zaraibi goats.

## Conclusion

The major finding of this study was that the methylation patterns of GDF-9 and GHR were markedly affecting the PMY and TMY in goats. In addition, many non-genetic factors significantly influence the productivity of milk in goats. Breeding season significantly affects milk production, where March had the greatest effect on PMY. Single birth does produce higher TMY than multiple ones. Additionally, Maximum milk production was attained in the third and fourth parity, that interactive effects should be considered when studying individual performance.

## Data Availability

Data are available upon request.
